# Transcript and proteomic analysis of developing white lupin (*Lupinus albus *L.) roots

**DOI:** 10.1186/1471-2229-9-1

**Published:** 2009-01-05

**Authors:** Li Tian, Gregory J Peel, Zhentian Lei, Naveed Aziz, Xinbin Dai, Ji He, Bonnie Watson, Patrick X Zhao, Lloyd W Sumner, Richard A Dixon

**Affiliations:** 1 Plant Biology Division, Samuel Roberts Noble Foundation, Ardmore, OK 73401, USA; 2 Department of Plant Sciences, University of California, Davis, Davis, CA 95616, USA; 3 CNAP, Department of Biology, University of York, York, YO 10 5YW, UK

## Abstract

**Background:**

White lupin (*Lupinus albus *L.) roots efficiently take up and accumulate (heavy) metals, adapt to phosphate deficiency by forming cluster roots, and secrete antimicrobial prenylated isoflavones during development. Genomic and proteomic approaches were applied to identify candidate genes and proteins involved in antimicrobial defense and (heavy) metal uptake and translocation.

**Results:**

A cDNA library was constructed from roots of white lupin seedlings. Eight thousand clones were randomly sequenced and assembled into 2,455 unigenes, which were annotated based on homologous matches in the NCBInr protein database. A reference map of developing white lupin root proteins was established through 2-D gel electrophoresis and peptide mass fingerprinting. High quality peptide mass spectra were obtained for 170 proteins. Microsomal membrane proteins were separated by 1-D gel electrophoresis and identified by LC-MS/MS. A total of 74 proteins were putatively identified by the peptide mass fingerprinting and the LC-MS/MS methods. Genomic and proteomic analyses identified candidate genes and proteins encoding metal binding and/or transport proteins, transcription factors, ABC transporters and phenylpropanoid biosynthetic enzymes.

**Conclusion:**

The combined EST and protein datasets will facilitate the understanding of white lupin's response to biotic and abiotic stresses and its utility for phytoremediation. The root ESTs provided 82 perfect simple sequence repeat (SSR) markers with potential utility in breeding white lupin for enhanced agronomic traits.

## Background

Nitrogen and phosphate are essential plant mineral nutrients and limiting factors for plant growth under stress. Due to poor soil conditions and limited nutrient uptake capacities, most crop plants require fertilizer applications to prevent nitrogen and phosphate deficiency; fertilizer use is expensive and causes serious long term ecological problems. It is therefore desirable to improve the efficiency of plant mineral nutrient uptake from soil. White lupin fixes nitrogen efficiently through its symbiotic association with Bradyrhizobia, and adapts to phosphate deficiency by developing cluster roots and secreting organic acids to solubilize inorganic phosphate in the soil [1]. In recent years, efforts have been directed toward understanding the mechanisms of nutrient uptake in white lupin for broader applications in crop improvement.

In addition to poor soil nutrient content, the use of lands for farming is also limited by metal contamination. There has been emerging interest in the use of white lupin for phytoremediation. White lupin can accumulate Zn, Mn, and Al, and heavy metals such as Cd, Pb, Hg and Cr, at high concentrations without affecting plant growth [2-4]. Although (heavy) metal uptake from soil and transport from root to shoot have been demonstrated in white lupin, little is known about the genes and enzymes responsible for (heavy) metal uptake and translocation within white lupin plants.

A wide variety of isoflavones are synthesized and exuded during development of white lupin roots. These include genistein and 2'-hydroxy genistein, and their 6-, 8-, and 3'-monoprenylated, 6, 3'-diprenylated, and 7-*O*-glucosyl derivatives. Isoflavones are well known for their roles in plant disease responses [5]. In contrast to the phenylpropanoid phytoalexins that accumulate upon pathogen attack, prenylated isoflavones accumulate constitutively in white lupin and are designated as phytoanticipins [6]. Prenylation significantly increases the activity of the core compounds, and prenyltransferase genes therefore have potential applications in plant disease resistance and human health [7]. Biochemical studies in white lupin have shown that more than one membrane-bound prenyltransferase is responsible for the prenyl transfer reactions, which occur at different positions of the isoflavone ring structure. Although the reactions leading to genistein and 2'-hydroxygenistein biosynthesis have been elucidated in several plant systems, the molecular identities of the isoflavone prenyltransferases are still unknown. Recently, a flavonoid specific prenyltransferase, naringenin 8-prenyltransferase (SfN8DT-1), was cloned and characterized from *Sophora flavescens *[8]. SfN8DT-1 is membrane-bound and is related evolutionarily to the previously identified plant aromatic prenyltransferases involved in tocopherol and plastoquinone biosynthesis [8].

The adenosine triphosphate-binding cassette (ABC) family transporters mediate transport of a wide variety of molecules across biological membranes and play critical roles in plant growth and development. Plant ABC transporters can be classified into 13 subfamilies based on their size, orientation, and the transmembrane and linker domains of the protein [9]. Transport of glycoside- and glutathione-conjugated phenylpropanoid compounds has been reported to be mediated by members of the multidrug resistance associated protein (MRP) subfamily of ABC transporters [10,11]. Recently, the involvement of an ABC transporter in the secretion of genistein aglycone from soybean roots was reported [12]. However, the mode of transportation of prenylated isoflavonoids remains unclear.

White lupin is an agronomically important crop because the grains are high in protein and fiber and low in starch and oil [13]. Understanding white lupin root development and metabolism will facilitate breeding for favorable agronomic traits. Furthermore, understanding the mechanism of (heavy) metal uptake and transport, and antimicrobial isoflavone synthesis and exudation in white lupin roots will have broad applications for understanding and engineering efficient soil nutrient uptake, disease resistance and phytoremediation properties in other plants. However, previous studies on white lupin root transcripts have focused on the gene expression patterns in cluster root development and in response to phosphate deficiency (in particular, the phosphate transporters) [14-17]. Most of the white lupin nucleotide sequences currently available in the GenBank database were obtained from two cDNA libraries that were constructed from cluster roots of phosphate-deficient white lupin plants [17]. White lupin still lacks extensive genomics resources. Furthermore, the steady state transcript levels in a specific organ such as roots may not directly reflect the corresponding protein population.

We here describe an EST database and a protein reference map from developing white lupin roots to aid identification of candidate genes relevant to antimicrobial defense and phytoremediation. The EST collection also revealed a number of simple sequence repeat (SSR) markers of potential utility for lupin breeding.

## Methods

### Plant material and RNA extraction

White lupin seeds (*Lupinus albus *v Lupro 2085) were scarified, germinated in a coarse sand and turface mixture and grown under greenhouse conditions (with a 16 h day, 8 h night cycle). Seedling roots were harvested at 5, 10, 15 and 20 days post-emergence, immediately frozen in liquid nitrogen and stored at -80°C prior to analysis. Total RNA was extracted from the root tissue using TRI reagent (Molecular Research Center, Cincinnati, OH). Equal amounts of RNA sample from each time point were pooled and the combined total RNA was used for cDNA library construction. Roots for protein analysis were grown under the same greenhouse conditions, and harvested at 17 days post-emergence. Normal roots (not cluster roots) were used for RNA extractions and protein analysis.

### cDNA library construction, EST sequencing and analysis

A developing white lupin root cDNA library was prepared from total RNA using the Creator Smart cDNA library construction kit (BD Bioscience, Palo Alto, CA) following the manufacturer's instructions, and was transformed into *E. coli*. Eight thousand bacterial clones were randomly picked and used for inoculation of liquid culture. Plasmid DNA was purified from the overnight liquid culture and sequenced with the M13 forward primer. After removing short reads (less than 100 bp) and vector sequences, the remaining ESTs were clustered and assembled with the TGICL program  using its default parameter settings (at least 40 bp overlap with at least 94% identities). The unigenes (contigs and singletons) were used for performing homology searches with an automatic BLAST search and data mining tool [18]. Six-frame translations of the unigene sequences were searched against the NCBI protein database using the BLASTX algorithm. Unigene sequences with *E *values greater than 0.1 were classified as no hit. For gene ontology (GO) analysis, the lupin root unigenes were searched against the *Arabidopsis thaliana *database  and the corresponding Arabidopsis Gene Index (AGI) numbers were obtained. AGI numbers of the best matches were used for searching the Arabidopsis GO annotations  and were divided into the three major classes that are common to all eukaryotes: biological process, cellular component and molecular function. The functional classification was also manually inspected and minor adjustments were made to ensure appropriate assignment of GO annotations.

### Protein extraction and gel electrophoresis

Roots of 17 d old white lupin seedlings were harvested and frozen in liquid nitrogen. The frozen root tissue was ground into a fine powder using a cryogenic grinder (Fisher Scientific, Hampton, NH). Two volumes of extraction buffer (100 mM potassium phosphate pH 7.5, 400 mM sucrose, 28 μM β-mercaptoethanol) and 5% polyvinylpolypyrrolidone (PVPP) were added to the ground powder. The root tissue was homogenized in the extraction buffer with a polytron twice for 30 s each, and then centrifuged at 8,000 g for 15 min. The supernatant was filtered through glass wool to remove the cell debris. Protein concentration was determined by the Bradford assay [19]. A portion of the supernatant was brought to a final concentration of (w/v) 12.5% trichloroacetic acid (TCA) with TCA/acetone solution and protein was collected by centrifugation. The protein pellet was then washed three times with 80% acetone containing 0.05% β-mercaptoethanol, air dried, resuspended in resolubilization solution (8 M urea, 4% CHAPS, 20 mM DTT, 0.2% biolytes), and analyzed by 2-D gel electrophoresis. The remainder of the supernatant was centrifuged at 140,000 g for 60 min. The microsomal pellet was washed twice with extraction buffer, resuspended in SDS-PAGE sample loading buffer, and analyzed by 1-D gel electrophoresis.

One thousand two hundred μg total proteins from developing white lupin roots were separated by 2-D gel electrophoresis as previously described [16]. One hundred μg microsomal proteins were separated in a pre-cast SDS-PAGE gradient gel (4–15%) and stained with Coomassie Brilliant Blue R250 (BioRad Laboratories, Hercules, CA). Gel images were captured by a BioRad Fluor S MultiImager (BioRad Laboratories, Hercules, CA).

### Protein digestion and identification

Protein spots from the Coomassie stained 2-D gel were manually excised, destained and in-gel digested with trypsin as previously described [20]. Peptide fragments from digested proteins were mixed (1:1, v/v) with matrix (α-cyano-4-hydroxycinnamic acid, 10 mg/ml), spotted and analyzed by matrix-assisted laser desorption/ionization-time of flight mass spectrometry (MALDI-TOF MS). Mass spectra were acquired using a PerSeptive Biosystems Voyager DE STR (Applied Biosystems, Framingham, MA) in the positive ion reflector mode with a mass range from 900 Da to 4,000 Da. One hundred shots were triggered for each spectrum and four independent spectra were collected and averaged. Protein identification through peptide mass fingerprinting (PMF) was performed by querying the peptide masses against both NCBInr protein database and a custom legume database developed by Dr. Lloyd Sumner's group and the Noble Foundation bioinformatics team, using Mascot that employs a probability-based scoring system (version 2.2, Matrix Science, UK). The custom legume database contains protein sequences imported from the NCBInr protein database for a range of legumes, including *Medicago sativa*, *M. truncatula*, *Lotus japonicus*, *Glycine max*, *Pisum sativum*, *Phaseolus vulguris*, *Lupinus albus *and *L. luteus *(Lei et al. unpublished). In addition, tentative contig (TC) sequences from *M. truncatula*, *L. japonicus *and *G. max*, and six-frame translations of developing white lupin root unigenes from the EST analysis were added to the custom legume database. For peptide matching, a maximum of one missed cleavage was allowed and a minimum of four peptide matches was required. The maximum molecular weight discrepancy was set at 100 ppm.

For microsomal proteins, sixteen 5 mm slices were excised from the 1-D protein gel and transferred to microcentrifuge tubes. After in gel trypsin digestion, peptides were extracted, separated and analyzed by LC-MS/MS. The quadruple-time of flight mass spectrometer QSTAR (Applied Biosystems, Forster City, CA) was coupled to a nano-scale high performance liquid chromatography (HPLC) system (Dionex, San Francisco, CA), which includes an autosampler (Famos), a precolumn switching device (Switchos) and an HPLC pump system (Ultimate), and allows for sensitive detection on small amounts of peptide samples. The HPLC gradient for peptide separation and the parameters for MS data acquisition and analysis were described previously [21]. Peptide identification was performed using the Mascot software (Matrix Science, Boston, MA). Both NCBInr protein database and the custom legume database were queried using the acquired mass spectral data, with a mass tolerance of 100 ppm. Proteins with a molecular weight search score greater than 30 and a minimum two matched peptides are reported.

### Mining of SSRs

SSR identification, PCR primer design and *in silico *amplification from the designed primers were performed and hosted by an in-house developed web server, PhpSSRMiner, which is available at . PhpSSRMiner provides a user-friendly interface which integrates its back-end pipeline to streamline the process of perfect SSR identification by SSRIT [22], imperfect SSR identification by Sputnik [23], PCR primer design using Primer3 [24] and *in silico *amplification of the designed primers via IsPCR [25]. The 2,455 white lupin unigenes were analyzed using the PhpSSRMiner web server to identify perfect SSR candidates with SSR length equal or greater than 20 nt. The analysis results can be exported from PhpSSRMiner as tab-delimited text files with separate columns for the positions and sequences of the forward and reverse primers, type of SSR repeat (perfect vs. imperfect), SSR motif and length.

## Results and discussion

### Generation and functional annotation of developing white lupin root ESTs

A total of 8,000 clones were randomly picked and sequenced from their 5' ends. After removing low quality, short reads and vector sequences, the remaining ESTs (5,150) were assembled into 2,455 unigenes, which included 540 contigs and 1,915 singletons. As of April 2008, 112 protein sequences and 2,412 nucleotide sequences from white lupin were present in the NCBI database, many from plants subjected to phosphate deficiency. The deposition of the developing white lupin root ESTs (GenBank accession numbers FG089554 – FG094703) has therefore doubled the amount of publicly available white lupin nucleotide sequences. In addition, 1544 (63%) of the developing white lupin root unigenes do not have any hits when searched against the previously published white lupin nucleotide sequences (tBLASTx search with an *E *value greater than or equal to 10^-5^), presenting new information on the white lupin genome (Additional File 1).

**Figure 1:**
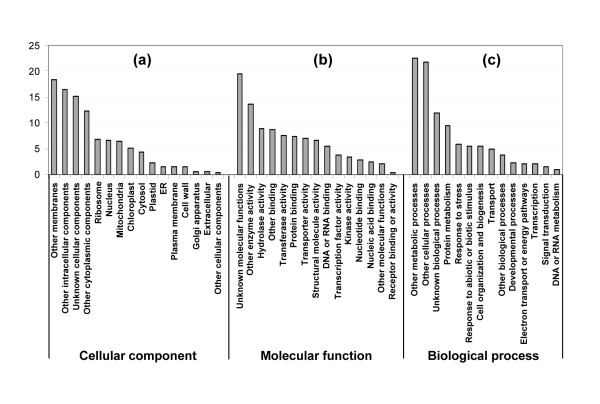
**Functional classification of the developing white lupin root unigene dataset**. The unigene sequences were searched against the Arabidopsis database  and were functionally classified based on the GO annotations of their Arabidopsis homologs. The relative frequencies (%) of GO hits (Y axis) for functional categories of a) cellular component, b) molecular function, and c) biological process are shown.

GO terms are commonly used to describe the functions of genes and gene products and to facilitate queries among genes from different organisms. A concise GO annotation requires combined computational and manual methods following a common standard that has been set by the Gene Ontology Consortium [26,27]. A detailed GO annotation system has been established for the fully sequenced *Arabidopsis thaliana *genome [28]. The white lupin root unigenes were functionally classified based on similarities with annotated genes in Arabidopsis. A total of 1,785 white lupin unigenes had significant hits (*E *value less than or equal to 10^-4^) with Arabidopsis genes. The relative frequency of white lupin root unigene hits in each GO category is presented in Figure 1. It should be noted that when the unigenes are described as unknown, they have unknown GO terms rather than representing genes encoding proteins with unknown functions.

The white lupin root unigenes cover a broad range of functional categories (Figure 1). Overall, the unclassified ("unknown" or "other") GO annotations accounted for over 40% of the total unigenes in each category. In the category of molecular function, 44% were classified as having unknown molecular functions, other enzyme activities, other binding and other molecular functions. In biological process, 60% of the unigenes were classified as other metabolic processes, other cellular processes, unknown biological processes and other biological processes. Under the category of cellular component, the four most frequent GO hits were other membranes, other intracellular components, unknown cellular components and other cytoplasmic components, accounting for a total of 63% in this category. In addition, although the white lupin ESTs were derived from root tissue, some unigenes showed sequence homology to Arabidopsis genes encoding chloroplast proteins, presumably due to certain conserved motifs that are shared between the sequences. Alternatively, it is possible that they are homologous sequences encoding chloroplast and leucoplast proteins, respectively. Overlapping annotations also existed among some of the subcategories.

Out of the top 20 most abundant unigenes from white lupin roots, 17 showed high similarity to genes encoding proteins from other leguminous plants, and 10 overlapped with the abundant unigenes in phosphate-deficient white lupin roots (Table 1) [17]. The most abundant unigene was a perfect match to the previously reported pathogenesis-related protein 10 from white lupin. LaPR-10 was previously isolated and characterized from 10 d old healthy root tissues of white lupin [29]. It showed sequence similarity to pathogenesis related proteins from other plant species and exhibited ribonuclease activity. Although most pathogenesis related proteins are induced upon pathogen attack, LaPR-10 is constitutively expressed throughout root development. The expression of LaPR-10 indicates that, together with the constitutive secretion of prenylated isoflavones, white lupin roots contain a well established, pre-formed defense system. Interestingly, a unigene with homology to pathogenesis-related protein 1 has the most redundant ESTs in a cDNA library derived from roots of phosphate-deficient common bean (*Phaseolus vulgaris*) plants [30].

**Table 1:** The 20 most abundantly expressed unigenes in the developing white lupin root cDNA library.

Unigene	# ESTs	Top BLAST hit	NCBI Accession No.	*E *value
0016	244	Pathogenesis-related 10 (*Lupinus albus*)	BAB63949	5e-84

0007	97	No hit		

0019	83	Ripening related protein (*Glycine max*)	AAD50376	8e-51

0021	30	Thiamin biosynthetic enzyme (*Glycine max*)	BAA88228	2e-154

0022	29	MtN5 (*Medicago truncatula*)	CAA75593	5e-23

0025	29	Hypothetical protein (*Medicago truncatula*)	ABD32999	0.013

0026	26	Profucosidase (*Pisum sativum*)	CAA09607	1e-67

0023	20	Probable aquaporin TIP-type (MtAQP1) (*Medicago truncatula*)	Q9FY14	1e-116

0027	19	No hit		

0028	19	Metallothionein-like protein (*Arachis hypogaea*)	AAO92264	4e-23

0030	17	Probable glutathione S-transferase (*Glycine max*)	P32110	4e-96

0031	16	Hypothetical protein (*Plantago major*)	CAH59408	8e-34

0035	15	Auxin-repressed protein (*Robinia pseudoacacia*)	AAG33924	3e-38

0038	14	Aquaporin (*Lupinus albus*)	CAA11025	1e-158

0041	12	Calcium-binding EF-hand (*Medicago truncatula*)	ABE82233	5e-41

0042	12	Aquaporin 2 (*Samanea saman*)	AAC17529	5e-149

0043	11	Thaumatin-like protein PR-5b (*Cicer arietinum*)	CAA09228	2e-126

0045	11	S-adenosylmethionine synthetase (*Medicago truncatula*)	ABO84685	1e-100

0047	11	Ubiquitin-like protein (*Pisum sativum*)	ABN05665	5e-44

0048	11	Major intrinsic protein (*Medicago truncatula*)	ABE81405	7e-98

Table 1 also shows the presence of abundant unigenes associated with metal and water uptake functions. When the second most abundant unigene was searched against the NCBI protein database, no significant homologs were found. However, when the NCBI non-redundant EST database was searched using the tBLASTx method, the unigene showed significant but low homology to a *Medicago sativa *type 1 metallothionein (MET1) (*E *= 1e-12). Metallothioneins are cysteine-rich proteins that can bind metals and oxidant radicals. Another white lupin homolog of metallothionein contained 19 ESTs in the present collection (Table 1). Aquaporins facilitate water transport across membranes, and several unigenes homologous to aquaporins were abundantly represented in the white lupin root EST database (Table 1). Genes encoding metallothioneins and aquaporins are also among the most abundant transcripts in non-stressed poplar (*Populus trichocarpa *× *deltoids*) roots [31]. Thiamine (vitamin B1) is used as a co-enzyme for dehydration of α-keto acids, such as in the reaction catalyzed by malate dehydrogenase, and is also a cofactor for pyruvate dehydrogenase. A unigene encoding a thiamin biosynthetic enzyme was the fourth most abundant unigene in the developing white lupin root cDNA library (Table 1).

### Isoflavonoid biosynthetic pathway genes are well represented in the EST database

**Figure 2:**
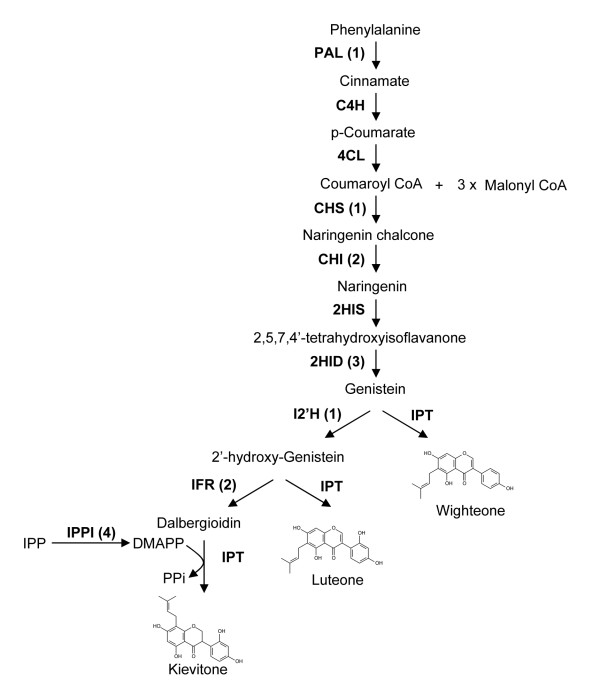
**Biosynthetic steps leading to prenylated isoflavonoids in white lupin**. Chemical structures of selected prenylated isoflavonoids are shown. Enzymes that catalyze the biochemical reactions are shown, with the corresponding number of identified white lupin root ESTs indicated in parentheses. PAL, L-phenylalanine ammonia-lyase; C4H, cinnamate 4-hydroxylase; 4CL, 4-coumarate:CoA ligase; CHS, chalcone synthase; CHI, chalcone isomerase; 2HIS, 2-hydroxyisoflavanone synthase; 2HID, 2-hydroxyisoflavanone dehydratase; I2'H, isoflavone 2'-hydroxylase; IFR, isoflavone reductase; IPPI, isopentenyl diphosphate/dimethylallyl diphosphate isomerase; IPT, isoflavone prenyltransferase.

Consistent with the constitutive synthesis and secretion of isoflavone metabolites, genes encoding isoflavone biosynthetic enzymes were present in the white lupin root EST database. These included the general phenylpropanoid pathway genes encoding L-phenylalanine ammonia-lyase, chalcone synthase, chalcone isomerase (isoforms 2 and 4), and the isoflavonoid pathway specific genes encoding 2-hydroxyisoflavanone dehydratase, isoflavone 2'-hydroxylase and isoflavone reductase (Figure 2). Flavonoid 4'-*O*-methyltransferase was also found in the lupin EST collection (Additional File 2).

In prenylated isoflavone biosynthesis, a prenyl (dimethylallyl pyrophosphate; DMAPP) group is attached to the core isoflavone molecule, catalyzed by a prenyltransferase. DMAPP is synthesized from its allylic precursor isopentenyl pyrophosphate (IPP) through either the cytosolic mevalonate pathway and or the plastidial 2-*C*-methyl-D-erythritol-4-phosphate (MEP) pathway [32]. Unigenes encoding enzymes of both the plastidial MEP pathway, including 1-deoxy-D-xylulose-5-phosphate reductoisomerase and 4-hydroxy-3-methylbut-2-enyl diphosphate synthase, and the cytosolic mevalonate pathway (3-hydroxy-3-methylglutaryl-CoA reductase) were present in the white lupin root cDNA library (data not shown). Two unigenes homologous to the UbiA family prenyltransferases were also found and may encode the isoflavone prenyltransferase(s) for prenylated isoflavone synthesis in white lupin (Additional File 2; Figure 2).

ABC transporters have been implicated in transport and root exudation of plant secondary metabolites [11,33]. Previous data also suggested that multiple ABC transporters could be involved in transporting a single group of metabolites [33]. Three ABC transporter homologs, including one MRP subfamily transporter, were identified in the white lupin root EST collection (Table 2) and may potentially be involved in secretion of isoflavone metabolites.

**Table 2:** White lupin root unigenes that are homologous to ATP binding cassette family transporters.

Unigene	# ESTs	BLAST hit	NCBI Accession No.	*E *value
1888	1	ABC transporter homolog (*Populus nigra*)	BAA94511	4e-80

1858	1	ATMRP1 (*Arabidopsis thaliana *multidrug resistance-associated protein 1); xenobiotic-transporting ATPase	NP_174329	4e-30

1682	1	ABC-2; ABC transporter related (*Medicago truncatula*)	ABO81333	3e-07

### Metal uptake, transport and phosphate uptake

Previous studies have focused primarily on the physiology of (heavy) metal uptake and transport in white lupin, but the process is not well understood at the molecular level. The present study identified 11 unigenes that were homologous to (heavy) metal transport proteins, copper chaperones and metal-transporting P-type ATPase from other plant species, but which had not been reported previously from white lupin (Table 3). Two unigenes showed high similarity to *Medicago truncatula *ZRT- and IRT-like proteins (ZIP), MtZIP1 and MtZIP4, respectively. MtZIPs were shown to functionally complement yeast metal-uptake defective mutants [34]. Specifically, MtZIP1 and MtZIP4 could restore the growth of mutant yeast in Zn- and Mn-limited media, respectively, suggesting that the two unigenes may encode Zn and Mn transporters in white lupin roots. Two unigenes were homologous to soluble copper chaperones that are responsible for intracellular distribution of copper ions [35]. Metal transporting ATPases interact with metallochaperones and function in transmembrane transport of metals. A homolog of metal transporting ATPase, PAA1, was present in the cDNA library. In Arabidopsis, PAA1 interacts with copper chaperones and transports Cu across the plastid envelope [36]. Six white lupin root unigenes contained heavy metal associated domains, suggesting roles of the encoded proteins in heavy metal transport or detoxification [37].

**Table 3:** White lupin root unigenes that are homologous to metal binding/transport proteins.

Unigene	# ESTs	BLAST hit	NCBI Accession No.	*E *value
2019	1	Metal transport protein, ZIP4 (*Medicago truncatula*)	AAR08414	6e-67

1902	1	Metal transport protein, ZIP1 (*Medicago truncatula*)	AAR08412	8e-60

0187	3	Copper chaperone homolog CCH (*Glycine max*)	AAF15286	1e-37

1528	1	Copper chaperone (*Populus alba *× *Populus tremula *var. glandulosa)	AAT12488	3e-30

0570	1	Metal-transporting P-type ATPase (*Arabidopsis thaliana*)	CAA20565	1e-10

1804	1	Heavy-metal-associated domain-containing protein/copper chaperone (CCH)-related (*Arabidopsis thaliana*)	NP_192597	4e-56

0413	2	Heavy-metal-associated domain-containing protein/copper chaperone (CCH)-related (*Arabidopsis thaliana*)	NP_197247	4e-53

0797	1	Heavy metal transport/detoxification protein (*Medicago truncatula*)	ABE84676	5e-61

0980	1	Heavy metal transport/detoxification protein (*Medicago truncatula*)	ABE90818	6e-31

1141	1	Heavy metal transport/detoxification protein (*Medicago truncatula*)	ABE90826	2e-27

2409	1	Heavy metal transport/detoxification protein (*Medicago truncatula*)	ABE93893	8e-25

Malate and citrate secretion to soil is observed from white lupin plants grown under low phosphate. Malate dehydrogenase, 2-oxoglutarate/malate translocator, citrate lyase and citrate synthase are key enzymes for citrate and malate metabolism and unigenes encoding these proteins were found in the cDNA library. These genes have also been implicated in aluminum tolerance [38]. Furthermore, several families of transcription factors, including MYB, WRKY, APETALA2 (AP2) and bHLH, have been implicated in the regulation of plant responses to phosphate starvation [39]. In addition, MYB and bHLH transcription factors are also involved in the regulation of metal uptake and transport [40-42]. Three bHLH, five WRKY, seven MYB and three AP2 transcription factors were represented in the developing white lupin root cDNA library and may function in phosphate and metal ion uptake and transport (Additional File 3).

### Characterization of SSR markers

Microsatellites or simple sequence repeats (SSRs) are tandemly repeated DNA sequences (in most cases 1–6 bases in length) highly abundant throughout prokaryotic and eukaryotic genomes [43]. Microsatellite markers are extremely informative, mostly co-dominant, and show simple Mendelian inheritance over repeated generations. The repeat elements in a motif are usually di-, tri-, tetra- or penta-nucleotides, and the number of blocks for each repeat element can be highly variable even among closely related individuals of the same species.

The white lupin EST collection was examined for SSR sequences by PhpSSRMiner. From the 2,455 unigenes, 82 sequences (3.34%) exhibited perfect SSRs, with 61 different motifs. The top 10 motifs are summarized in Additional File 4, and represent a diverse set of sequence types. Primer sequences for amplifying the SSR motifs were designed using PhpSSRMiner, which could potentially be used by plant breeders in marker-assisted selection applications (Additional File 5).

### A protein reference map of developing white lupin roots

**Figure 3:**
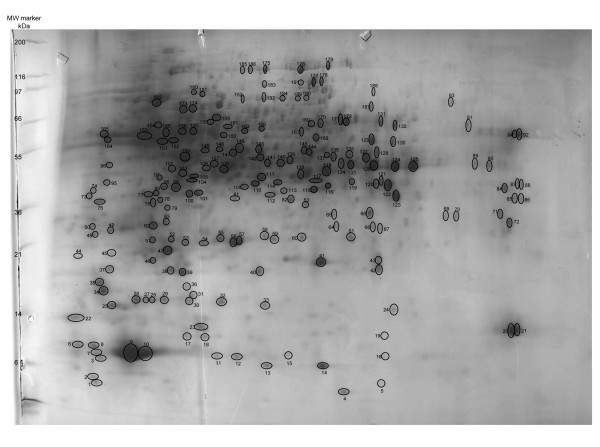
**2-D reference map of total proteins extracted from developing white lupin roots**. One thousand two hundred μg proteins were separated by IEF (pH 3–11) and SDS-PAGE (12% vertical gel) and stained with Coomassie blue R-250. One hundred and ninety proteins were selected (indicated by numbered spots) across the PI and molecular mass range, in-gel digested with trypsin and subjected to MALDI-TOF peptide mass fingerprinting.

To provide a more comprehensive understanding of white lupin root biology, we generated a reference map of total proteins extracted from developing roots. One thousand two hundred μg proteins were separated by isoelectric focusing on a nonlinear gradient of pH 3 to 11 according to their isoelectric point, and were subsequently separated in a second dimension on a 12% SDS-PAGE gel according to their molecular mass (Figure 3). A total of 190 protein spots were selected across the pI and molecular mass range and were excised from the 2-D gel. The protein spots were digested with trypsin and the eluted peptides subjected to PMF using a MALDI-TOF mass spectrometer. High quality peptide mass spectra were obtained for 170 protein spots.

The automated Mascot search engine  was used to identify protein sequences in the NCBInr database based on mass spectra. Searches against this database did not yield significant matches according to the Mascot protein scores, defined as -10*log(P), where P is the probability that the observed match is a random event. The Mascot probability-based scoring system for PMF takes into account the abundance of homologous proteins in the target database and relies on the database size. The NCBInr database contains more than 5 million protein sequences derived from over 5,000 organisms. The overall low protein scores observed are possibly due to the relatively low abundance of white lupin protein homologs in the large NCBInr database. A target database that is small in size, but "enriched" in white lupin protein homologs, was needed to improve the confidence of protein identifications.

A custom legume database was therefore constructed and searched to facilitate the identification of white lupin proteins (Lei et al., unpublished). The legume database contains protein sequences from several leguminous species, namely *Medicago sativa*, *M. truncatula*, *Lotus japonicus*, *Glycine max*, *Pisum sativum*, *Phaseolus vulguris*, *Lupinus albus *and *L. luteus*. Six-frame translations of the white lupin unigene sequences from the EST analysis were also included in the legume database. Furthermore, tentative contig (TC) sequences of *M. truncatula*, *G. max *and *L. japonicus *were translated into protein sequences and added to the legume database. TCs are assembled from overlapping ESTs, thus containing longer and more useful sequence information than the underlying ESTs. TC sequences have previously been used to enhance protein identification success rate in *M. truncatula *[20,21]. Out of the 170 analyzed protein spots, 21 could be matched confidently (Mascot protein score > 75) to sequences in the legume database. Fourteen additional protein spots had Mascot protein scores between 50 and 75, but the majority of peaks in the spectra overlapped with the matched peptides, suggesting a confident match. Peptide information and Mascot protein scores of the 35 identified proteins are provided in Additional File 6. Though in most cases only one protein match was found for each protein spot, two distinct proteins were identified for protein spot 61 (Additional File 6).

The above proteomic analysis provided further insight into biological function and metabolism of developing white lupin roots. Proteins involved in ATP production were present, including triosephosphate isomerase and glyceraldehyde 3-dehydrogenase in glycolysis and NADH dehydrogenase in the mitochondrial respiratory complex. Ascorbate peroxidase is a critical component of the reactive oxygen species (ROS) scavenging system. Cytosolic ascorbate peroxidase is essential for plant defense responses [44] and was found in the white lupin root total proteins. Proteins involved in flavonoid, fatty acid and amino acid metabolism were abundant: including chalcone synthase and dihydroflavonol 4-reductase for flavonoid biosynthesis; β-ketoacyl synthase that adds malonyl-ACP to fatty acids for chain elongation; serine hydroxymethyltransferase that catalyzes the reversible conversion of serine and glycine; and glutamine synthetase that catalyzes the condensation of glutamate and ammonia to form glutamine, a process that assimilates ammonia and is important in nitrogen metabolism.

### Membrane proteins from developing white lupin roots

**Figure 4:**
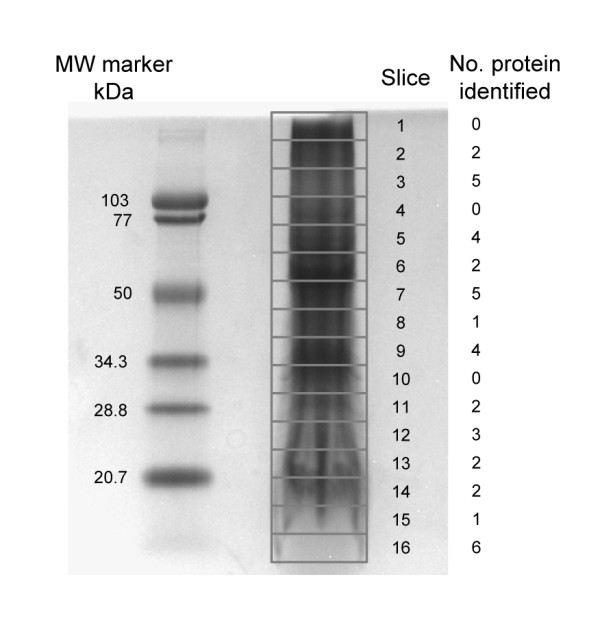
**1-D SDS-PAGE gel of developing white lupin root microsomal proteins**. One hundred μg microsomal proteins were solubilized in SDS-PAGE sample buffer and separated on a 4–15% gradient polyacrylamide gel. The protein gel was stained with Coomassie blue R-250. Sixteen 5 mm slices were excised from the 1-D gel as outlined. The gel slices were digested with trypsin and subjected to LC-MS/MS analysis.

Membrane proteins are typically underrepresented in 2-D gel analyses using total proteins because of protein precipitation caused by the hydrophobic domains [45]. A 1-D gel approach was therefore undertaken to identify membrane proteins from developing white lupin roots. Following electrophoresis, the SDS-PAGE gel was cut into sixteen 5 mm slices (Figure 4). After in-gel trypsin digestion, peptides were extracted from each gel slice, passed through a cation exchange column and three fractions were collected: flow-through, 500 mM and 1 M ammonium acetate eluates, respectively. An aliquot of each fraction was loaded onto a reverse phase HPLC column and analyzed by LC-MS/MS. In contrast to the PMF method for protein identification, LC-MS/MS analysis obtains the protein sequence information by fragmentation of individual peptides. A total of 39 unique proteins were identified by two or more peptides (Table 4).

**Table 4:** List of membrane proteins from developing white lupin roots identified by LC-MS/MS.

Protein annotation	Score	Protein Mass (Da)	# Peptides	%Coverage
Adenosine triphosphatase (*Phaseolus vulgaris*)	518	55310	12	27.4

Adenine nucleotide translocator (*Lupinus albus*)	345	42134	8	23.7

F1 ATPase (*Pisum sativum*)	310	60113	7	13.9

Adenine nucleotide translocator (*Lupinus albus*)	117	39769	5	15.7

AAA ATPase (*Medicago truncatula*)	243	167308	4	2.8

ATPase beta subunit (*Nicotiana sylvestris*)	188	22337	4	25

Plasma membrane H+ ATPase (*Lupinus albus*)	106	105034	4	4.2

Trans-cinnamic acid 4-hydroxylase (*Pisum sativum*)	104	57023	4	8.8

60S ribosomal protein L22-2 (RPL22B) (*Arabidopsis thaliana*)	148	20969	3	17.5

Histone H4 (*Hyacinthus orientalis*)	106	20687	3	16.7

H^+^-transporting two-sector ATPase (*Medicago truncatula*)	92	33840	3	12.3

DDT; Homeodomain-related (*Medicago truncatula*)	40	19112	3	13.8

Hypothetical protein (*Medicago truncatula*)	123	20972	2	9.6

PDR-like ABC-transporter (*Glycine max*)	112	162565	2	1.3

Heat shock protein Hsp70 (*Medicago truncatula*)	111	61346	2	4.3

BiP-isoform D (*Glycine max*)	104	54230	2	5.1

60S ribosomal protein (*Medicago sativa*)	95	20286	2	12.8

Mitochondrial phosphate transporter (*Glycine max*)	91	39765	2	4.8

60S ribosomal protein L12 (*Capsicum annuum*)	90	22609	2	13.9

Clathrin heavy chain (*Glycine max*)	90	193232	2	1.2

Aquaporin (*Lupinus albus*)	84	45880	2	6.8

Putative ATP synthase (*Arabidopsis thaliana*)	84	22787	2	9

Clathrin propeller, N-terminal (*Medicago truncatula*)	80	193039	2	1.2

Glycoprotein-like protein (*Solanum tuberosum*)	79	22873	2	9.4

Vacuolar ATP synthase catalytic subunit A (Vacuolar proton pump subunit alpha) (*Vigna radiata var. radiate*)	77	23405	2	8

H+ transporting ATPase, proton pump (*Medicago truncatula*)	76	104871	2	2.6

Ubiquinol-cytochrome C reductase complex 14 kDa protein, putative (*Arabidopsis thaliana*)	72	25275	2	10.2

40S ribosomal protein S14 (*Lupinus luteus*)	71	22413	2	13.2

Orn/DAP/Arg decarboxylase 2; Heat shock protein Hsp70 (*Medicago truncatula*)	70	22803	2	11.8

60S ribosomal protein L23a (*Daucus carota*)	64	26851	2	9.6

Ly200 protein (*Capsicum annuum*)	61	24171	2	9.1

Ribosomal protein L13a (*Lupinus luteus*)	56	23496	2	11.7

Glycoprotein-like protein (*Solanum tuberosum*)	56	26610	2	9.5

Trans-cinnamate 4-monooxygenase (C4H) (*Medicago sativa*)	53	58243	2	3.6

Putative ATP synthase subunit (*Glycine max*)	51	20242	2	12.3

Translation elongation factor 1A-5 (*Gossypium hirsutum*)	51	64778	2	3.3

Probable aquaporin PIP-type 7a (Turgor-responsive protein 7a) (*Pisum sativum*)	41	26029	2	9.2

Ubiquitin extension protein (*Lupinus albus*)	39	23489	2	10.8

Unnamed protein product (*Vitis vinifera*)	34	23564	2	6.7

Mitochondrial and vacuolar ATPases were highly abundant in the membrane fraction (Table 4). ATPases are often transmembrane proteins. The dephosphorylation of ATP to ADP releases energy and is coupled to import of metabolites for cellular and enzymatic functions and export of toxins and wastes. Consistent with their representation in the EST dataset, ABC transporters and aquaporins, which assist in water passage through membranes, were also found in the membrane proteome analysis (Tables 1 and 4).

Two characteristic ER-localized proteins were found in the microsomal fraction of developing white lupin roots. The chaperone BiP is a luminal binding protein in the ER. The molecular weight of this protein in *Glycine max *is 104 kD. Its white lupin homolog was separated on the 1-D gel in the molecular weight range of 70 kD to 103 kD (Figure 4, gel slice 4). Cinnamate 4-hydroxylase (C4H) is a membrane-bound cytochrome P450 enzyme that participates in the early steps of phenylpropanoid pathway metabolism and has been shown to be bound to ER-membranes [46]. Two white lupin root microsomal proteins, homologous to *Medicago sativa *and *Pisum sativum *C4H, respectively, migrated around the 50 kD size marker on the 1-D gel (Figure 4, gel slice 7; Table 4). Although the two white lupin C4H proteins had similar molecular mass, they were eluted at 1 M and 500 mM ammonium acetate, respectively, suggesting that they have different ionic charge properties. Endomembrane and plasmamembrane proteins, such as glycoproteins potentially involved in cell signaling and recognition, ribosomal proteins and heat shock proteins potentially involved in protein synthesis, were also quite abundant in the membrane fraction.

## Conclusion

Using genomic and proteomic approaches, we have identified candidate genes and proteins involved in (heavy) metal binding and transport, and antimicrobial isoflavonoid biosynthesis and transport, in white lupin roots. Further biochemical characterization of the candidate genes is needed to unequivocally delineate their functions *in planta*. The combined EST and protein datasets from developing white lupin root will not only contribute to our understanding of the response of white lupin to environmental stresses, but will also have broad application in the study of plant nutrient uptake, disease resistance and phytoremediation. Furthermore, our data provide a basis for future comparative studies between developing roots and the cluster roots that are formed under phosphate deficiency. Finally, the EST collection has been mined for SSR markers that will be potentially valuable for marker assisted selection of important agronomic traits in lupin. These markers could also enhance marker density in the white lupin linkage map (developed using amplified fragment length polymorphisms [AFLPs] and candidate gene-based markers) [47].

## Authors' contributions

LT carried out the cDNA library and PMF analyses, performed protein extractions, and drafted the manuscript. GJP carried out the 2-D gel and PMF analyses, and performed protein extractions. ZL carried out the LC-MS/MS analysis. NA generated the cDNA library. DX, JH and PZ performed the bioinformatic analyses on the ESTs. BW carried out the 2-D gel analysis. LWS assisted in the protein data analysis. RAD conceived of the study, directed the experimentation, and assisted in the preparation of the manuscript. All authors read and approved the final manuscript.

## Supplementary Material

Additional file 1 **Developing white lupin root unigene BLAST results**. BLAST search results of developing white lupin root unigenes against the NCBI database. Click here for file

Additional file 2 **Genes with homology to phenylpropanoid biosynthetic pathway enzymes**. Developing white lupin root unigenes that are similar to phenylpropanoid biosynthetic pathway enzymes from other plants based on the deduced amino acid sequences. Click here for file

Additional file 3 **Putative transcription factors in the white lupin root cDNA library**. Developing white lupin root unigenes encode putative transcription factors. Click here for file

Additional file 4 **The top 10 motifs in the SSR markers from the white lupin root cDNA library**. The most common and distinguishable SSR motifs identified in the developing white lupin root cDNA library. Only perfect SSR markers with a minimum length of 20 nucleotides were scored. Click here for file

Additional file 5 **Primer sequences for amplifying white lupin SSR motifs**. SSR motifs that were identified from the developing white lupin root ESTs and primer sequences that were designed using PhpSSRMiner for amplification of these white lupin SSRs. Click here for file

Additional file 6 **Proteins identified in developing white lupin roots by MALDI-TOF mass spectrometry**. A summary of white lupin root proteins identified by peptide mass fingerprinting. Click here for file
