# Supplementary material for: Transcript and proteomic analysis of developing white lupin (Lupinus albus L.) roots
Source: BMC Plant Biol. 2009 Jan 5;9:1. doi: 10.1186/1471-2229-9-1 (PMC2630931; doi:10.1186/1471-2229-9-1)
Supplement: Additional file 2 — Genes with homology to phenylpropanoid biosynthetic pathway enzymes. Developing white lupin root unigenes that are similar to phenylpropanoid biosynthetic pathway enzymes from other plants based on the deduced amino acid sequences. [file 1471-2229-9-1-S2.doc]

**Additional File 2. Genes with homology to phenylpropanoid biosynthetic pathway enzymes.**

| Unigene | # ESTs | BLAST hit | NCBI  Accession No. | *E* value |
| --- | --- | --- | --- | --- |
| 1352 | 1 | Phenylalanine ammonia-lyase (*Lotus japonicus*) | BAF36972 | 2e-90 |
| 2132 | 1 | Chalcone and stilbene synthases (*Medicago truncatula*) | ABE82745 | 2e-37 |
| 2251 | 1 | Putative chalcone isomerase 2 (*Lotus japonicus*) | BAC53984 | 6e-76 |
| 0968 | 1 | Putative chalcone isomerase 4 (*Glycine max*) | AAT94362 | 1e-69 |
| 0271 | 3 | 2-Hydroxyisoflavanone dehydratase (*Glycine max*) | BAD80840 | 2e-62 |
| 0826 | 1 | Cytochrome P450 monooxygenase CYP81E10; isoflavone 2'-hydroxylase (*Glycine max*) | ABC68400 | 1e-83 |
| 1658 | 2 | Isoflavone reductase homolog Bet v 6.0101 (*Betula pendula*) | AAC05116 | 1e-91 |
| 1533 | 1 | Flavonoid 4'-*O*-methyltransferase (Mentha x piperita) | AAR09602 | 2e-16 |
| 0729 | 1 | UbiA prenyltransferase (*Medicago truncatula*) | ABO81303 | 4e-53 |
| 1782 | 1 | Homogentisate phytyltransferase VTE2-2 (*Glycine max*) | ABB70128 | 2e-08 |
