# Supplementary material for: Transcript and proteomic analysis of developing white lupin (Lupinus albus L.) roots
Source: BMC Plant Biol. 2009 Jan 5;9:1. doi: 10.1186/1471-2229-9-1 (PMC2630931; doi:10.1186/1471-2229-9-1)
Supplement: Additional file 3 — Putative transcription factors in the white lupin root cDNA library. Developing white lupin root unigenes encode putative transcription factors. [file 1471-2229-9-1-S3.doc]

**Additional File 3. Putative transcription factors in the white** lupin root cDNA library.

| Unigene | # ESTs | BLAST hit | NCBI  Accession No. | *E* value |
| --- | --- | --- | --- | --- |
| 1396 | 1 | Basic helix-loop-helix (bHLH) family protein (*Arabidopsis thaliana*) | NP_195498 | 3e-12 |
| 0765 | 1 | Basic helix-loop-helix (bHLH) family protein (*Arabidopsis thaliana*) | NP_563749 | 1e-46 |
| 0576 | 1 | Basic helix-loop-helix (bHLH) family protein (*Arabidopsis thaliana*) | NP_177590 | 5e-20 |
| 0670 | 1 | WRKY protein (*Solanum tuberosum*) | ABN69038 | 1e-49 |
| 0951 | 1 | Putative WRKY transcription factor 30 (*Vitis aestivalis*) | AAR92477 | 7e-37 |
| 1044 | 1 | WRKY22 (*Arabidopsis thaliana*) | NP_192034 | 2e-27 |
| 1614 | 1 | WRKY3 (*Oryza sativa*) | AAQ20903 | 3e-12 |
| 1785 | 1 | WRKY31(*Arabidopsis thaliana*) | NP_567644 | 5e-11 |
| 0514 | 2 | MYR1 (MYB-RELATED PROTEIN 1) (*Arabidopsis thaliana*) | NP_197325 | 4e-39 |
| 0975 | 1 | MYB transcription factor MYB122 (*Glycine max*) | ABH02922 | 2e-49 |
| 1229 | 1 | MYB transcription factor MYB50 (*Glycine max*) | ABH02824 | 9e-27 |
| 1415 | 1 | MYB DNA-binding domain-containing protein (*Musa acuminata*) | ABF70013 | 4e-14 |
| 1787 | 1 | MYB transcription factor MYB114 (*Glycine max*) | ABH02918 | 4e-34 |
| 2095 | 1 | MYB family transcription factor / ELM2 domain-containing protein (*Arabidopsis thaliana*) | NP_178446 | 9e-21 |
| 2455 | 1 | MYB transcription factor MYB122 (*Glycine max*) | ABH02922 | 2e-54 |
| 0477 | 2 | AP2 domain-containing transcription factor family protein (*Arabidopsis thaliana*) | NP_173609 | 2e-25 |
| 1085 | 1 | AP2 domain-containing transcription factor (*Arabidopsis thaliana*) | NP_174636 | 1e-30 |
| 2315 | 1 | AP2-related transcription factor (*Mesembryanthemum crystallinum*) | AAF63205 | 2e-27 |
