# Supplementary material for: Transcript and proteomic analysis of developing white lupin (Lupinus albus L.) roots
Source: BMC Plant Biol. 2009 Jan 5;9:1. doi: 10.1186/1471-2229-9-1 (PMC2630931; doi:10.1186/1471-2229-9-1)
Supplement: Additional file 4 — The top 10 motifs in the SSR markers from the white lupin root cDNA library. The most common and distinguishable SSR motifs identified in the developing white lupin root cDNA library. Only perfect SSR markers with a minimum length of 20 nucleotides were scored. [file 1471-2229-9-1-S4.doc]

**Additional File 4. The top 10 motifs in the SSR markers from the white lupin root cDNA library.** Only perfect SSR markers with a minimum length of 20 nucleotides were scored.

| Rank | Motif | Motif Length | # SSRs | # Distinct SSR Patterns |
| --- | --- | --- | --- | --- |
| 1 | TC | 2 | 4 | (TC)10 [1]    (TC)13 [1]    (TC)14 [1]    (TC)20 [1] |
| 2 | CAC | 3 | 3 | (CAC)7 [3] |
| 3 | CT | 2 | 3 | (CT)10 [2]    (CT)12 [1] |
| 4 | TCT | 3 | 3 | (TCT)9 [1]    (TCT)12 [2] |
| 5 | TTC | 3 | 3 | (TTC)7 [2]    (TTC)8 [1] |
| 6 | GA | 2 | 3 | (GA)11 [1]    (GA)12 [1]    (GA)13 [1] |
| 7 | CTT | 3 | 3 | (CTT)7 [1]    (CTT)8 [1]    (CTT)9 [1] |
| 8 | ATG | 3 | 2 | (ATG)7 [1]    (ATG)23 [1] |
| 9 | AAG | 3 | 2 | (AAG)9 [2] |
| 10 | GTG | 3 | 2 | (GTG)7 [1]    (GTG)8 [1] |
