# Supplementary material for: Transcript and proteomic analysis of developing white lupin (Lupinus albus L.) roots
Source: BMC Plant Biol. 2009 Jan 5;9:1. doi: 10.1186/1471-2229-9-1 (PMC2630931; doi:10.1186/1471-2229-9-1)
Supplement: Additional file 6 — Proteins identified in developing white lupin roots by MALDI-TOF mass spectrometry. A summary of white lupin root proteins identified by peptide mass fingerprinting. [file 1471-2229-9-1-S6.doc]

**Additional File 6. Proteins identified in developing white lupin roots by MALDI-TOF mass spectrometry.**

| Spot ID | Annotation | Score | # peptides | % coverage | *E* value | Mr | pI |
| --- | --- | --- | --- | --- | --- | --- | --- |
| 52 | Triosephosphate isomerase (*Arabidopsis thaliana*) | 55 | 7 | 40 | 0.13 | 19710 | 9.24 |
| 54 | Triosephosphate isomerase (*Glycine max*) | 62 | 8 | 48 | 0.031 | 25218 | 7.93 |
| 55 | Cytosolic ascorbate peroxidase (*Arachis hypogaea*) | 83 | 9 | 32 | 0.00022 | 38733 | 5.57 |
| 60 | Proteasome subunit alpha type 6 (20S proteasome alpha subunit A) | 61 | 9 | 44 | 0.037 | 21961 | 9.08 |
| 61a | Unnamed protein product (*Vitis vinifera*) | 97 | 13 | 72 | 8.3e-006 | 22656 | 8.94 |
| 61b | GTP-binding protein (*Helianthus annuus*) | 83 | 13 | 50 | 0.00023 | 33804 | 6.01 |
| 67 | Heat shock protein Hsp70 | 92 | 8 | 41 | 0.0011 | 22443 | 8.57 |
| 72 | KIF4-like, partial (8%) | 79 | 13 | 49 | 0.023 | 29307 | 9.77 |
| 84 | Uncoupling protein 1b (*Glycine max*) | 55 | 8 | 46 | 0.14 | 25677 | 9.63 |
| 87 | Ovarian tumour, otubain (*Medicago truncatula*) | 65 | 16 | 20 | 0.013 | 111992 | 8.72 |
| 94 | eEF-1a (*Glycine max*) | 58 | 13 | 27 | 0.069 | 49347 | 9.14 |
| 116 | Homolog to At2g01140 (*Arabidopsis thaliana*) | 101 | 14 | 30 | 0.00014 | 59652 | 9.25 |
| 117 | NADH dehydrogenase subunit 11 | 77 | 10 | 30 | 0.033 | 41012 | 8.54 |
| 119 | TGF-beta receptor-interacting protein 1 (*Phaseolus vulgaris*) | 63 | 10 | 32 | 0.02 | 35854 | 6.84 |
| 123 | Guanine nucleotide-binding protein subunit beta-like protein | 89 | 11 | 42 | 6e-05 | 35586 | 7.62 |
| 124 | glyceraldehyde-3-dehydrogenase C subunit (*Glycine max*) | 81 | 10 | 34 | 0.00034 | 33801 | 7.27 |
| 125 | glyceraldehyde-3-dehydrogenase C subunit (*Glycine max*) | 81 | 10 | 34 | 0.00034 | 33801 | 7.27 |
| 126a | glyceraldehyde-3-dehydrogenase C subunit (*Glycine max*) | 100 | 12 | 37 | 4.5e-06 | 49541 | 7.22 |
| 126b | glyceraldehyde-3-dehydrogenase C subunit (*Glycine max*) | 75 | 9 | 39 | 1.4e-03 | 33801 | 7.27 |
| 129 | Dihydroflavonol 4-reductase (*Lotus japonicus*) | 54 | 7 | 27 | 0.17 | 34472 | 6.37 |
| 130 | Beta-ketoacyl synthase (*Medicago truncatula*) | 53 | 10 | 28 | 0.23 | 49879 | 8.05 |
| 131 | Serine hydroxymethyltransferase (Serine methylase) (Glycine hydroxymethyltransferase) (SHMT) | 82 | 12 | 20 | 0.012 | 68796 | 8.81 |
| 132 | SHM4 (Serine hydroxymethyl transferase 4); glycine hydroxymethyltransferase (*Arabidopsis thaliana*) | 62 | 8 | 38 | 0.028 | 22387 | 5.49 |
| 133 | Glyceraldehyde-3-dehydrogenase C subunit (*Glycine max*) | 86 | 10 | 30 | 0.00012 | 49541 | 7.22 |
| 137 | Chalcone synthase (*Lupinus albus*) | 117 | 16 | 48 | 8.7e-08 | 40131 | 6.35 |
| 139 | Alcohol-dehydrogenase (*Glycine max*) | 85 | 10 | 30 | 9.8e-04 | 36357 | 6.13 |
| 140 | Alcohol-dehydrogenase [Glycine max]. | 64 | 9 | 23 | 0.017 | 36357 | 6.13 |
| 142 | Glutamine synthetase leaf isozyme, chloroplast precursor (Isozyme delta) (Glutamate--ammonia ligase). (*Phaseolus vulgaris*) | 63 | 10 | 29 | 0.02 | 47217 | 6.77 |
| 157 | CAB75428 | 70 | 10 | 28 | 0.0045 | 47755 | 5.14 |
| 163 | IMGA_AC150776_9.1_MTIMGAG HSP 70 | 103 | 16 | 29 | 8.8e-05 | 71046 | 5.11 |
| 166 | S-adenosyl-L-homocysteinase (*Lupinus luteus*) | 135 | 16 | 42 | 1.4e-09 | 53292 | 5.64 |
| 171 | IMP dehydrogenase/GMP reductase | 88 | 17 | 29 | 0.0028 | 70889 | 5.8 |
| 172 | atpA (*Glycine max*) | 147 | 17 | 45 | 8.7e-11 | 55296 | 6.23 |
| 179 | UP|ACOC_CUCMA (P49608) Aconitate hydratase | 113 | 23 | 22 | 8.8e-06 | 127463 | 6.81 |
| 182 | Cytosolic phosphoglucomutase (*Pisum sativum*) | 53 | 6 | 41 | 0.22 | 19837 | 4.97 |
